# Supplementary material for: An optogenetic assay of Drosophila larval motor neuron performance in vivo
Source: J Neurosci Methods. Author manuscript; Available in PMC 2026 May 18. (PMC13183466; doi:10.1016/j.jneumeth.2025.110661)
Supplement: Supplemental material [file NIHMS2171519-supplement-Supplemental_material.pdf]

All supplemental data, including .bonsai and .io files, can be found in this public GitHub repository: https://github.com/GabrielBonassi77/Motor-Performance-Assay
